# An updated and illustrated dichotomous key for the Chagas disease vectors of *Triatomabrasiliensis* species complex and their epidemiologic importance

**DOI:** 10.3897/zookeys.805.25559

**Published:** 2018-12-11

**Authors:** Carolina Dale, Carlos Eduardo Almeida, Vagner José endonça, Jader Oliveira, João Aristeu da osa, Cleber Galvão, Jane Costa

**Affiliations:** 1 Laboratório de Biodiversidade Entomológica, Instituto Oswaldo Cruz (IOC), FIOCRUZ, Brazil Laboratório de Biodiversidade Entomológica, Instituto Oswaldo Cruz Rio de Janeiro Brazil; 2 Instituto de Biologia, Universidade Estadual de Campinas, UNICAMP, Brazil Universidade Estadual de Campinas Camínas Brazil; 3 Universidade Federal do Piauí, UFPI, Brazil Universidade Federal do Piauí Piauí Brazil; 4 Faculdade de Ciências Farmacêuticas, Universidade Estadual Paulista “Júlio de Mesquita Filho”, FCFAR/UNESP, Brazil Universidade Estadual Paulista “Júlio de Mesquita Filho” São Paulo Brazil; 5 Laboratório Nacional e Internacional de Referência em Taxonomia de Triatomíneos, Instituto Oswaldo Cruz (IOC), FIOCRUZ, Brazil Instituto Oswaldo Cruz Rio de Janeiro Brazil

**Keywords:** kissing bugs, morphological key, species group

## Abstract

In the subfamily Triatominae, *Triatoma* exhibits the largest number of species, which are arranged in complexes. For the *T.brasiliensis* species complex, recent investigations based on results of geometric morphometrics combined with phylogeny have provided evidence that it should be composed of seven species: *T.brasiliensis, T.bahiensis*, *T.juazeirensis*, *T.lenti, T.melanica*, *T.petrocchiae*, and *T.sherlocki*, in which *T.brasiliensis* is divided in two subspecies: *T.b.brasiliensis* and *T.b.macromelasoma*. A taxonomic key is presented to identify each taxon. Among members of this complex, *T.b.brasiliensis* is the most important in an epidemiologic context, due to its high prevalence in natural infection by *Trypanosomacruzi* combined with a pronounced adaptation to domiciliary habitats. However, some members may be currently invading and colonizing homes, a process known as domiciliation. Therefore, the key presented here may be potentially useful for researchers as well as those involved in vector control measures.

## Introduction

Chagas disease is an infection caused by the etiologic agent *Trypanosomacruzi* (Chagas, 1909), a protozoan transmitted to humans and other mammals through the feces of infected hematophagous insects of subfamily Triatominae. Currently, this group consists of more than 150 species, of which more than 65 are found in Brazil (Costa and Lorenzo 2009; Galvão 2014; Oliveira and Alevi 2017; Oliveira et al. 2018). In addition to vector-borne transmission (the main route), several other modes are known, including congenital transmission, accidental intake of contaminated food (e.g., açai juice and sugarcane juice), organ transplantation, blood transfusion, breastfeeding, and laboratory accidents (WHO 2017). In the subfamily Triatominae, *Triatoma* exhibits the largest number of species, which are arranged in complexes, a kind of grouping that was initially based in varied rationalities, as ecology, geographic distribution, cytogenetics, among others (see Oliveira et al. 2017). Phylogenetic reconstruction based on multiple mitochondrial genes did not recover *Triatoma* as a monophyletic unit (Gardim et al. 2014; Justi et al. 2014). Despite this, it is a genus with several species of epidemiological importance, as *T.infestans* in several countries of South America, *T.dimidiata* in Central America and *T.brasiliensis, T. pseudomaculata* and *T.sordida* in Brazil (Coura 2015). Justi et al. (2014) presented a comprehensive phylogenetic study of Triatomini, proposing the species complexes should be composed by natural groups.

*Triatomabrasiliensis* species complex represents a monophyletic unit (Oliveira et al. 2017) and was first suggested as a group (Costa et al. 2013) based on data on morphology (Costa et al. 1997), biology (Costa and Marchon-Silva 1998), crossing experiments (Costa et al. 2003b), ecology (Costa et al. 1998; Almeida et al. 2009), isoenzymes (Costa et al. 1997), dispersal abilities (Almeida et al. 2012), and DNA variation analyses (Monteiro et al. 2004). Recent cytogenetic, morphological and molecular studies (Alevi et al. 2013, 2014, 2015, 2018; Mendonça et al. 2014, 2016; Oliveira et al. 2017) have shown that other species, in addition to those previously identified (Costa et al. 2013), should be included in the *T.brasiliensis* complex, which currently consists of *T.brasiliensis* Neiva, 1911, *T.bahiensis* Sherlock & Serafim, 1967, *T.juazeirensis* Costa & Felix, 2007, *T.lenti* Sherlock & Serafim, 1967, *T.melanica* Neiva & Lent, 1941, *T.petrocchiae* Pinto & Barreto, 1925, and *T.sherlocki* Papa et al., 2002, in which *T.brasiliensis* is divided in two subspecies *T.b.brasiliensis* Neiva, 1911 and *T.b.macromelasoma* Galvão, 1956.

To date, most of measures to combat the transmission of Chagas disease have been focused on vector control. Defining the taxonomic status and correctly identifying vectors of the *T.brasiliensis* complex is crucial to the success of surveillance actions, because each species exhibits its own epidemiological importance (Costa et al. 2003a, 2013). Because the *T.brasiliensis* species complex suffered rearrangements after phylogenetic and morphometric studies, an update of the dichotomous key by Costa et al. (2013) for members of the *T.brasiliensis* complex is proposed, according to the new consensus for defining this group.

## Materials and methods

Most of insects studied here are deposited in the Entomological Collection of Oswaldo Cruz Institute (**CEIOC**), Oswaldo Cruz Foundation, Rio de Janeiro, Brazil. The type species were always checked if possible, as previously detailed (Costa et al. 2013). For the newly included members (*T.petrocchiae*, *T.lenti*, and *T.bahiensis*) material from the insectary of Araraquara was also used that was deposited in the Dr Jose Maria Soares Barata Triatominae Collection (CTJMSB) of the São Paulo State University Julio de Mesquita Filho (UNESP), School of Pharmaceutical Sciences (FCFAR), Araraquara, São Paulo, Brazil. Insects from this insectary were also used for taking the photographs. The terminology of Lent and Wygodzinsky (1979) is followed.

## Results

According to Lent and Wygodzinsky (1979), the genus *Triatoma* comprises species in most cases with less than 30 mm. Other features include femora denticulate or not; ventral connexival plates distinct, although narrow in some cases. The sides of abdomen are rarely membranous, with membrane connecting dorsal and ventral connexival plates. The posterior process of pygophore is narrowly tapering apically. Members of *T.brasiliensis* species complex share a combination of characteristics: (i) they compose a natural group of (ii) inhabitants of rocky outcrops, (iii) distributed in semi-arid zones of Brazilian Northeast in the Caatinga Biome. The only exception is *T.melanica*, which can be also found in connections between Caatinga and Cerrado in the state of Minas Gerais. They are spread in many states, such as Bahia (**BA**), Ceará (**CE**), Maranhão (**MA**), Minas Gerais (**MG**), Paraíba (**PB**), Pernambuco (**PE**), Piauí (**PI**), and Rio Grande do Norte (**RN**).

A pictorial dichotomous key for the *T.brasiliensis* species complex was built up as follows:

**Table:** 

1	Brachypterous specimens (short wings for both genders), hemelytra not extending beyond the posterior margin of urotergite VI; legs unusually long; overall color dark brown to black, connexiva and femora with reddish orange markings	***T.sherlocki* (BA)**
	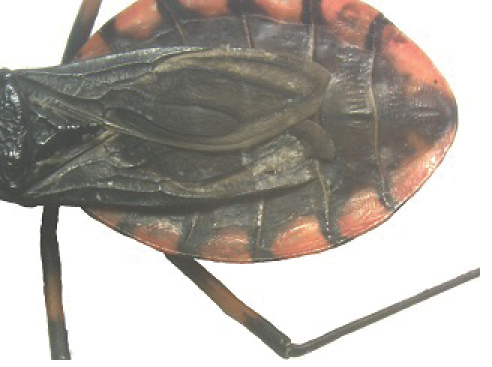	
–	Macropterous specimens, hemelytra reaching or almost reaching urotergite VII	**2**
	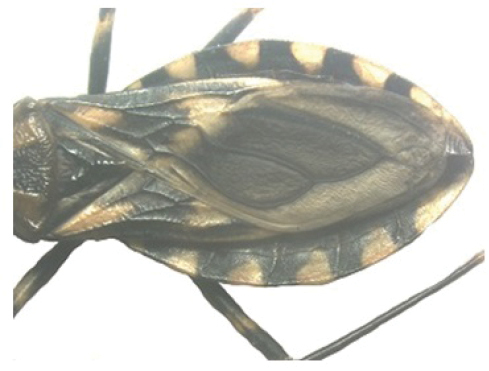	
2	Short first antennal segment, not close to reaching apex of clypeus	***T.petrocchiae* (BA, CE, PB, PE, RN)**
	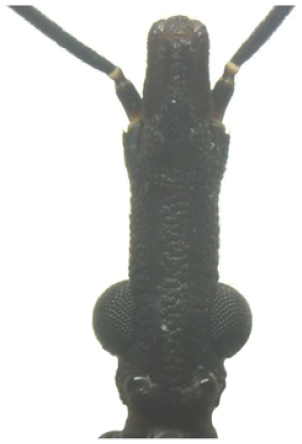	
–	First antennal segment reaching or almost reaching the level of apex of clypeus	**3**
	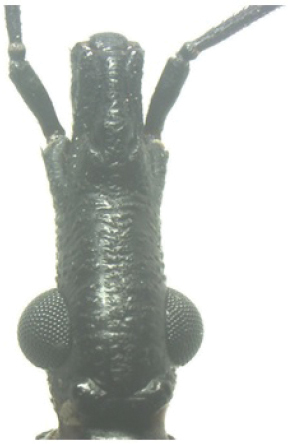	
3	Pronotum with 1+1 pale colored areas or stripes	**4**
	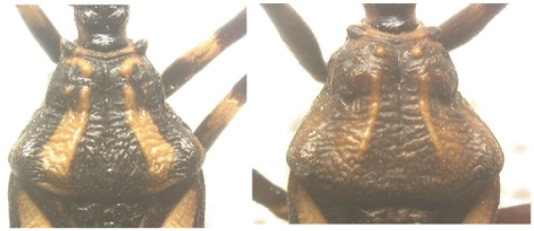	
–	Pronotum with entirely dark anterior lobe	**6**
	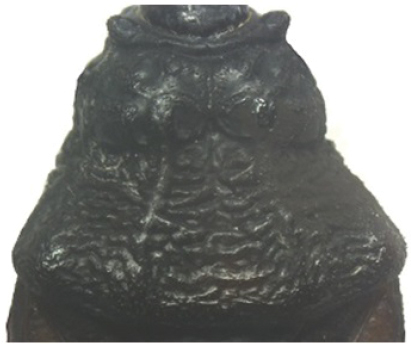	
4	Pronotum with 1+1 narrow brownish-yellow stripes; membrane of hemelytra with lumen of cells partially darkened	***T.b.macromelasoma* (PE)**
	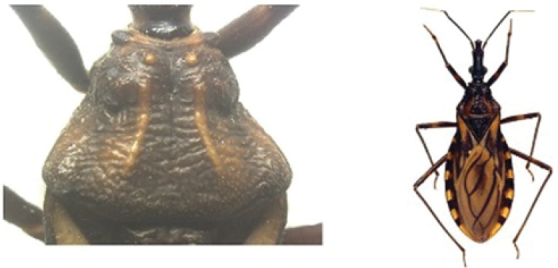	
–	Pronotum with 1+1 broad, elongated brownish yellow areas; membrane of hemelytra with lumen of cells entirely darkened or not	**5**
5	Pronotum with 1+1 brownish yellow areas extending from the posterior portion of anterior lobe to posterior lobe; femora with broad brownish yellow rings; membrane of hemelytra with lumen of cells not darkened; males with fossula spongiosa on fore tibia only	***T.b.brasiliensis* (CE, MA, PB, PI, RN)**
	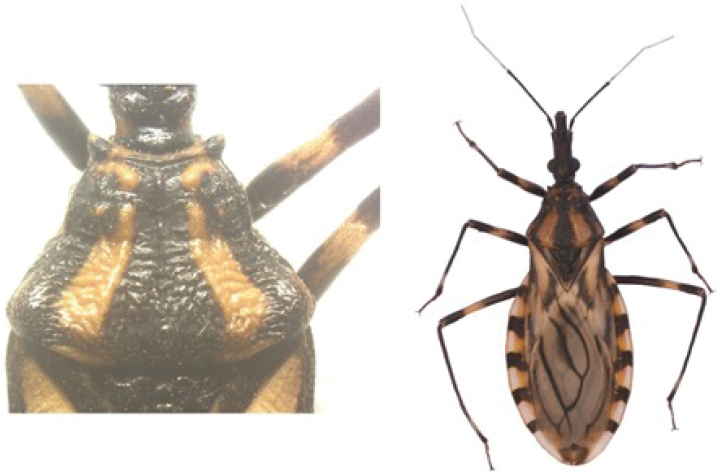	
–	Pronotum with 1+1 brownish yellow areas only on posterior lobe; femora with narrow brownish yellow rings; membrane of hemelytra with lumen of cells entirely darker; males with fossula spongiosa on fore tibiae	***T.melanica* (BA, MG)**
	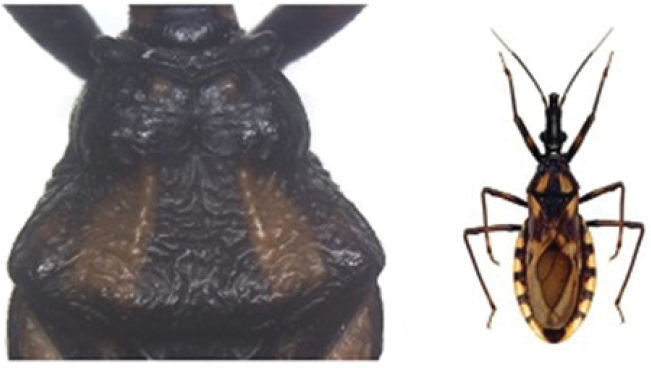	
6	Pronotum with black anterior lobe and wrinkled posterior lobe; rarely with few inconspicuous brownish yellow marks; light yellow corium with dark areas of variable extent; dark legs with light colored areas on trochanter	***T.juazeirensis* (BA)**
	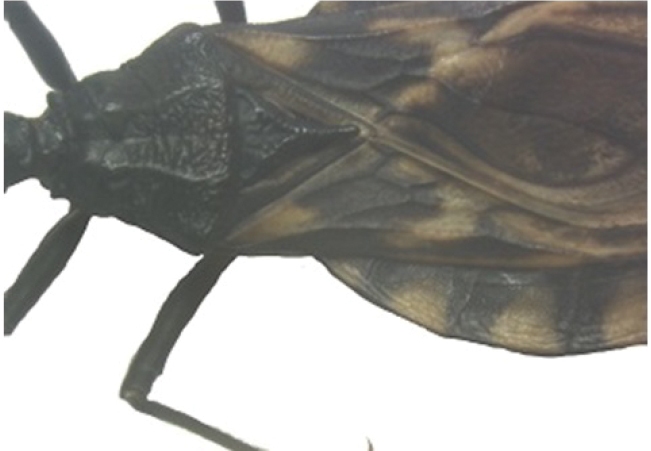	
–	Pronotum entirely black, non-granular, with anterolateral angles short and apically rounded; corium and clavus dark brown to black, and dark brown membrane; legs uniformly black	**7**
	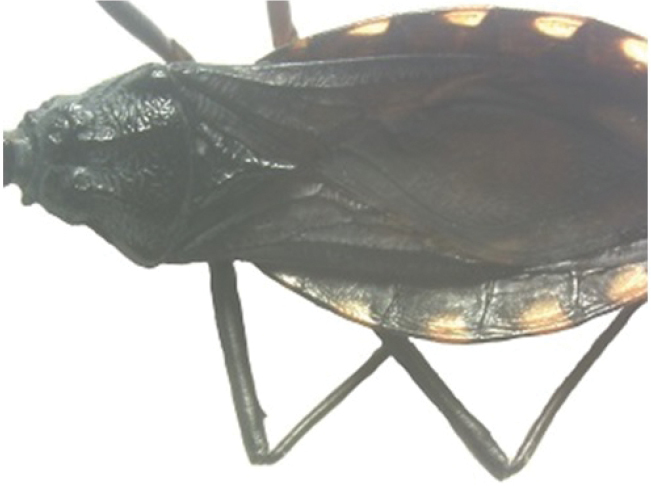	
7	Scutellum with posterior portion of central depression pointed; first abdominal segment without prominences; anterior region of prothorax (near the stridulatory sulcus) with a depression in ventral view; posterior region of stridulatory sulcus with rounded and well defined edges; mesothorax smooth and rounded	***T.lenti* (BA)**
–	Scutellum with posterior portion of central depression rounded; first abdominal segment with two lateral prominences; no depression on anterior region of prothorax; mesothorax with a central longitudinal projection, rectangular in shape	***T.bahiensis* (BA)**

## Discussion

The epidemiological profile of Chagas disease has changed, both in endemic areas and in other regions due to human migration, resulting in dissemination to countries where there is no vector transmission (Coura 2013; Dias et al. 2014). In Brazil, despite the control of *T.infestans*, the main Chagas disease vector in the past (Dias et al. 2002; Moncayo and Silveira 2017) each geographic region presents distinct challenges due to different ecological profiles of the distinct vectors and also due to dramatic environmental modifications. For instance, oral transmissions have been observed in the north region of Brazil (Coura 2013). In the south of Brazil, the persistent *T.rubrovaria* requires intensive monitoring actions (Almeida et al. 2002) and in the northeast region, species of the *T.brasiliensis* complex have been showing rapid changes in their behavior and ecology due to environmental anthropization (Costa et al. 2014). Therefore, in endemic areas, monitoring the synanthropic behavior of Chagas disease vectors is a challenge (Costa 1999; Costa and Lorenzo 2009). This can be illustrated by the case of *T.sherlocki*: a species described as sylvatic was later found invading and colonizing domiciles in a quarry mining community in a remote area of Gentio do Ouro, Bahia state (Almeida et al. 2009). Therefore, a comprehensive taxonomic key is crucial to be used by researchers and by those involved in vector control (Lent and Wygodzinsky 1979).

Members of the *T.brasiliensis* complex have been found in 12 Brazilian states and show mainly allopatric and parapatric distribution patterns, (Costa et al. 2003a, 2014; Gurgel-Gonçalves et al. 2012; Mendonca et al. 2016). However, some species of this complex are known to be occasionally found in sympatry, as *T.b.brasiliensis*, *T.b.macromelasoma*, and *T.juazeirensis* (Costa et al. 2014, 2016) which are all sympatric with the newly included *T.petrocchiae* (Oliveira et al. 2017), which renders geography alone as an imperfect tool for confidently identifying species. Other species may also be found later to be sympatric with each other. For instance, a hybrid zone between *T.b.brasiliensis* and *T.juazeirensis* was found (Costa et al. 2009, 2016), highlighting the utility of this key in detecting intermediate forms between them.

Studies on members of the complex have demonstrated that *T.b.brasiliensis* is the most important species in epidemiological terms. This species exhibits high intra-domiciliary infestation and infection rates (Costa et al. 2003a), which led Lilioso et al. (2017) to attribute a possible role to this species in a recent Chagas disease outbreak in Rio Grande do Norte State (Vargas et al. 2018). Additionally, via molecular markers, the existence of perennial and uncontrollable foci has been demonstrated in the sylvatic areas of populations with high *T.cruzi* prevalence (Almeida et al. 2008, 2016).

We recommend disseminating a version of this document in Portuguese to those involved in vector control measures. However, despite the contribution presented here, we still face some taxonomic challenges regarding this complex. There is no available key to differentiate immature stages for all members of this complex, which may complicate the correct identification of these forms. As mentioned above (Oliveira et al. 2017), some members are sympatric (e.g., *T.petrocchiae* and *T.brasiliensis*), and if immature forms of *T.petrocchiae* are found in domiciles, it may be operationally recorded as *T.brasiliensis* during regular vector inspections, because this last species is the most frequently found in domiciles wherever it occurs. Therefore, a comprehensive taxonomic key is a crucial tool for use by researchers and by those involved in vector control, which should include also immature forms.
